# Identifying and documenting osseous trauma from shark attacks by post mortem CT examination and autopsy

**DOI:** 10.1007/s12024-024-00823-6

**Published:** 2024-05-04

**Authors:** Roger W. Byard, Raj Perumal

**Affiliations:** Adelaide School of Biomedicine, The University of Adelaide, Forensic Science SA, Frome Rd and Divett Place, Adelaide, SA 5005 Australia

**Keywords:** Shark attack, Fatality, Forensic, CT scanning, 3-dimensional volume rendering, Silicone casting

## Abstract

A 15-year-old male was attacked by a large white shark while surfing. CT examination revealed an above-knee amputation of the right lower extremity with stripping of soft tissues from the groin distally. 3-dimensional volume rendering did not show any fragments of shark teeth but did reveal linear gouges, areas of shaving of cortical bone and an inverted ‘V’-shaped defect at the distal margin of the femoral shaft. At autopsy these injuries were confirmed in addition to areas with fine parallel cross-striations matching the marginal serrations of the teeth of a white shark. Thus, while post mortem CT with 3-dimensional reconstruction at high resolution can show the nature and number of the bony injuries following shark attack, it is complimented by pathological examination which may find fine parallel grooves from teeth serrations. Post mortem 3-dimensional volume rendering may also help to find or exclude fragments of teeth, and silicone casting may provide a permanent record of bone lesions.

## Introduction

Although shark fatalities are a source of great community and media interest, the occurrence of these deaths is put into some perspective by data from the United States which show that annually there are more than four times the number of fatalities caused by cows than sharks (22 vs. 5) [1]. The unexpected and public nature of many of these marine predator attacks, however, ensures that they are widely publicised, with sometimes lurid media, internet and film depictions adding to public concerns.

There has been a documented increase in numbers of cases over recent decades in certain locations [2–4]. The reasons for this are unclear but significant factors include increases in human populations near the sea with more time spent on activities such as surfing and less commonly diving [5–7]. Other factors may be reduction in water clarity and increases in water temperatures [8, 9]. Attacks may also be related to occupations such as fishing, with 4–6% of deaths in Australian fishers being attributed to sharks [10].

While international data on shark attacks are patchy, Australia is ranked as second in terms of numbers of bites annually, with an increase from nine bites per year from 1990 to 2000 to 22 bites per year from 2010 to 2020 [11]. Information on shark attacks in Australia is comprehensive with 1,196 cases documented between 1791 and 2020 listed in the Australian Shark-Incident Database. This was initiated in the 1980s as the Australian Shark Attack File and now is maintained by the Taronga Conservation Society Australia. There have been 297 fatalities reported in Australia with 26 of those occurring in waters around South Australia [11, 12].

Forensic and medical practitioners will in all likelihood be seeing more of these cases in future years, and so an understanding of the nature of these attacks and the types of injuries that may be encountered is required. As guides to autopsy approaches, injury assessment and tissue handling are available in the literature [13–15], the following report focusses instead on the pattern and morphology of bone trauma seen radiologically and at autopsy, and steps that may be taken to adequately document and/or preserve the features of such injuries.

## Case report

A 15-year-old male was surfing at a South Australian beach when he was attacked by a large white shark in front of witnesses. Although he managed to swim away from the shark and was pulled from the water, resuscitative attempts were unfortunately to no avail.

**Fig. 1:**
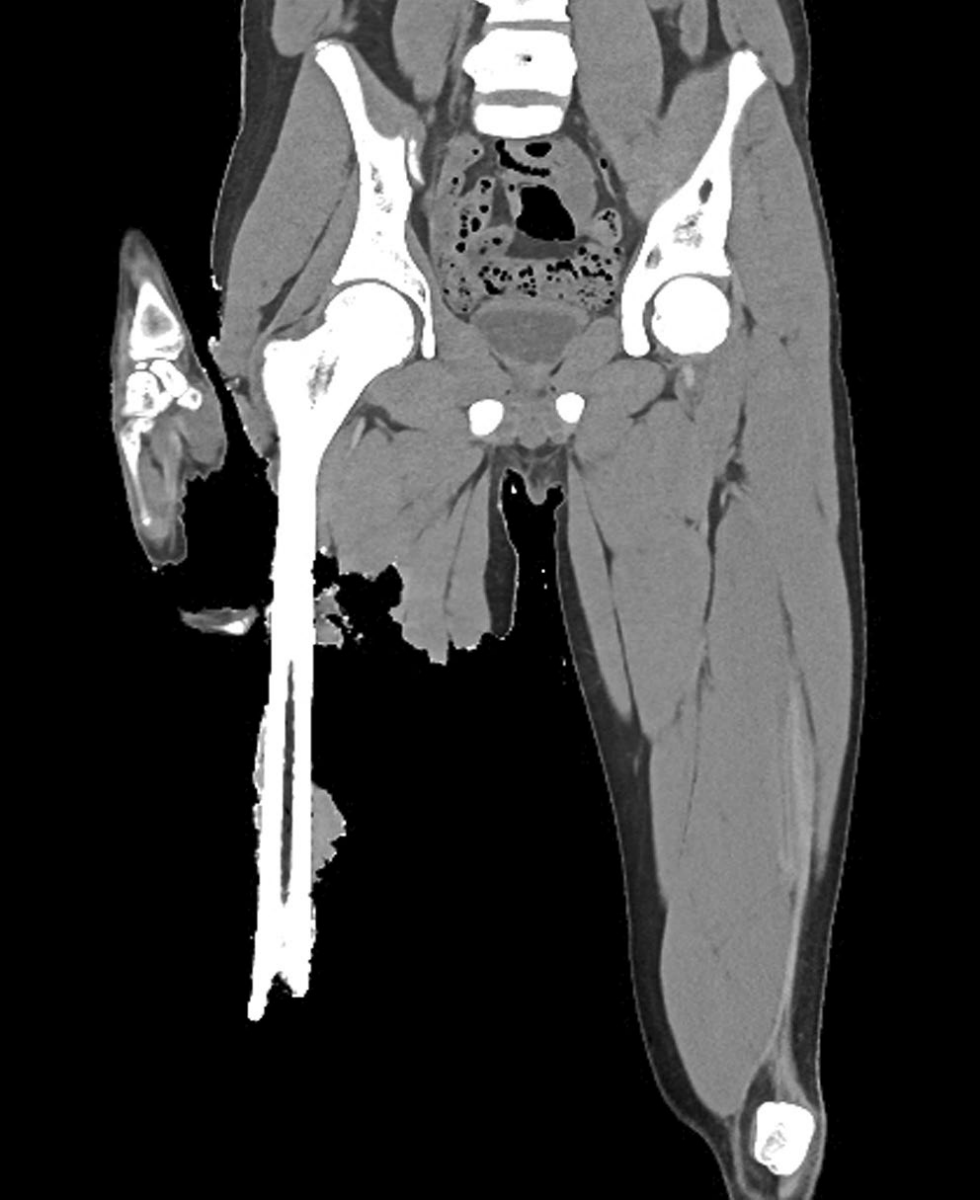
A post mortem CT scan showing loss of the distal femur and lower right leg along with extensive skin and soft tissue debridement

**Fig. 2:**
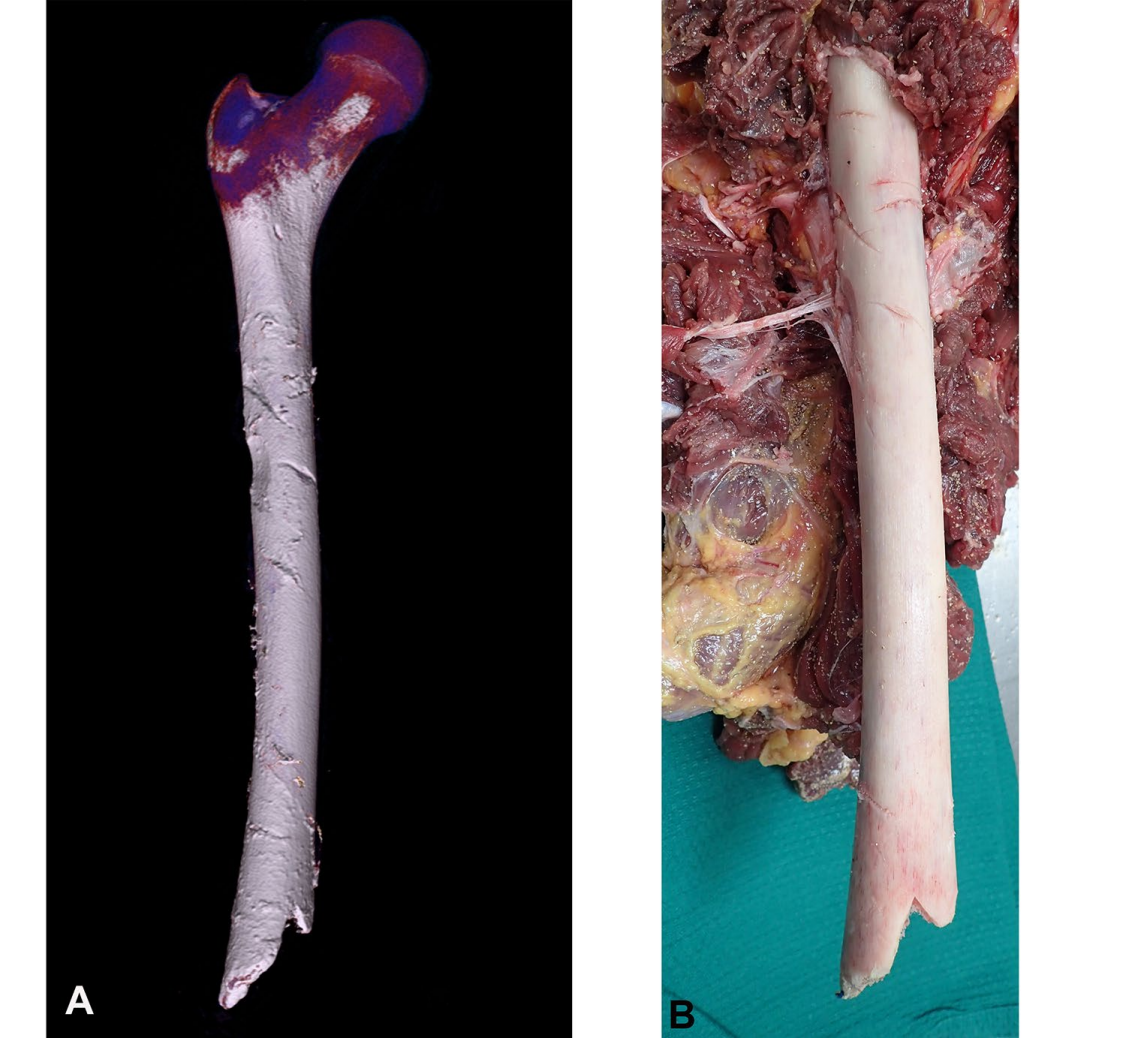
A 3-dimensional reconstruction of the femur at high resolution showing bone injuries more clearly (**A**). The residual right femur was exposed after overlying soft tissues had been removed by the shark. Osseous injuries ranged from amputation with a ‘V’ shaped bite mark, to shaving, gouging and scoring from the edges of the shark’s teeth (**B**)

An external examination with CT and toxicology was ordered by the State Coroner. CT examination revealed no abnormalities of the head, chest or abdomen. The major site of injury was the right lower extremity which had an above-knee amputation with stripping of soft tissues from the groin distally. In addition to routine CT examination (Fig. 1), high resolution image reconstruction of the right thigh was undertaken (0.5 mm slice thickness X 0.4 mm slice thickness overlap; 150 mm field of view; 512 matrix size; SAFIRE strength 3; Kernel Br46). 3-dimensional volume rendering was also performed to look for fragments of shark teeth (none were found). A number of bony defects were identified consisting of linear gouges, areas of shaving of cortical bone and an inverted ‘V’-shaped defect at the distal margin of the femoral shaft (Fig. 2A).

At autopsy the body was that of a young well-nourished male clothed in a wet suit which had the right leg torn off. He was around the stated age of 15 years with a height of 174 cm and a weight of 51 kg. As was already documented on CT examination the most significant injuries were to the right lower extremity, the lower portion of which was missing. The above knee amputation was associated with extensive skin, subcutaneous and soft tissue loss extending from an obliquely oval sharp-edged defect situated in the upper anterior right thigh running downward and medially to the inner right thigh and downward to the lateral aspect of the right buttock. Posteriorly the injury was less well defined with shredding of residual skin and skeletal muscle. A triangular skin defect was present posterolaterally measuring 90 × 15 mm. Anteriorly the severed end of the common femoral artery was identified. In addition, there was transection of the superficial femoral artery and a full thickness anterior defect in the profunda femoris artery.

**Fig. 3:**
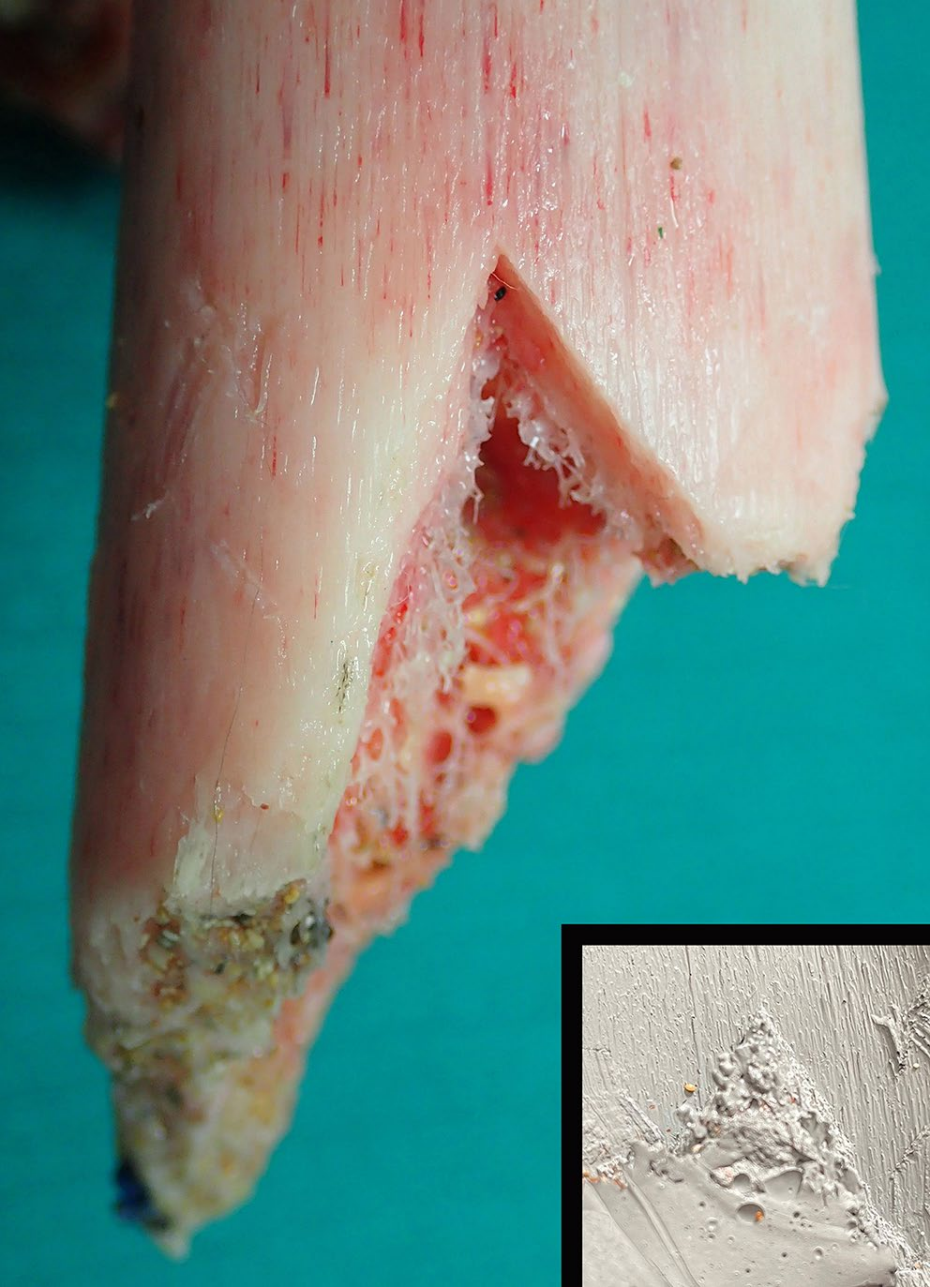
A closer view of the ‘V’ shaped bite mark at the edge of the fractured distal femur with a silicone cast recording the features of the injury (Inset)

**Fig. 4:**
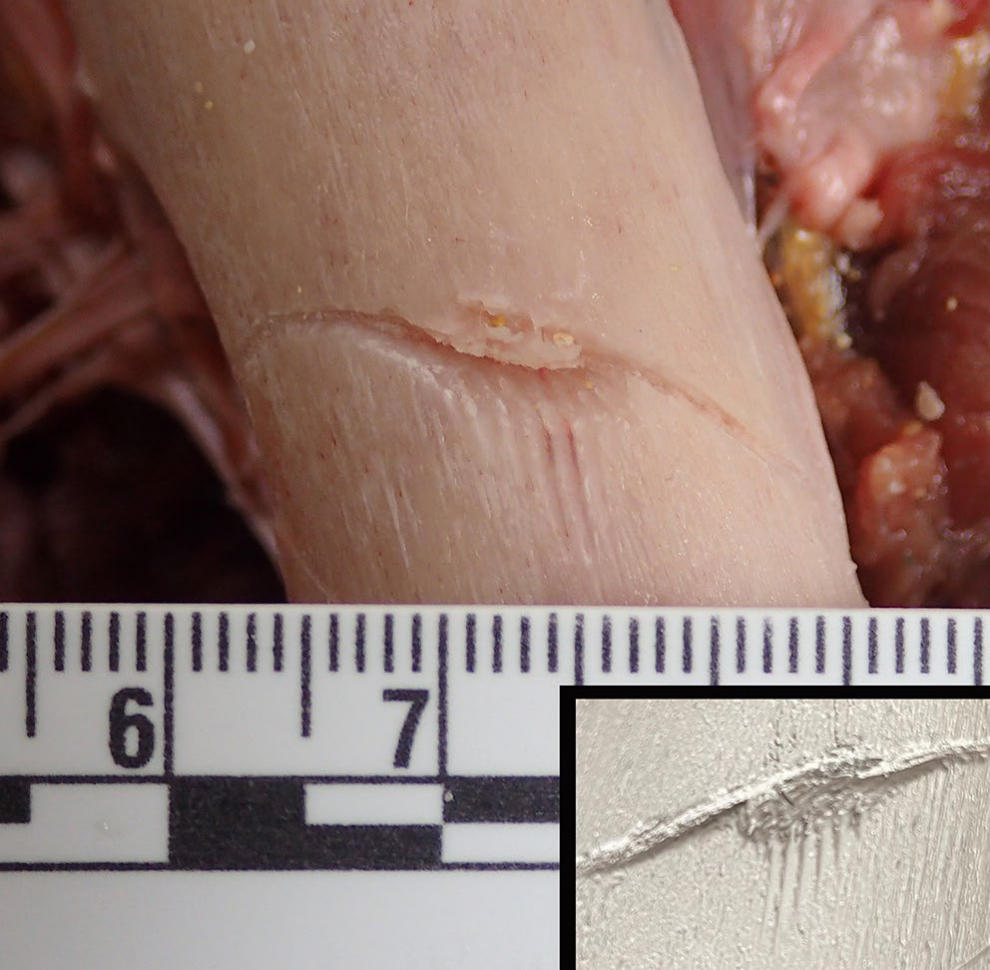
A gouged area of bone on the anterior aspect of the femur showing fine linear striations on the lower margin from the serrated edge of a shark tooth with a silicone cast recording the features of the injury (Inset)

**Fig. 5:**
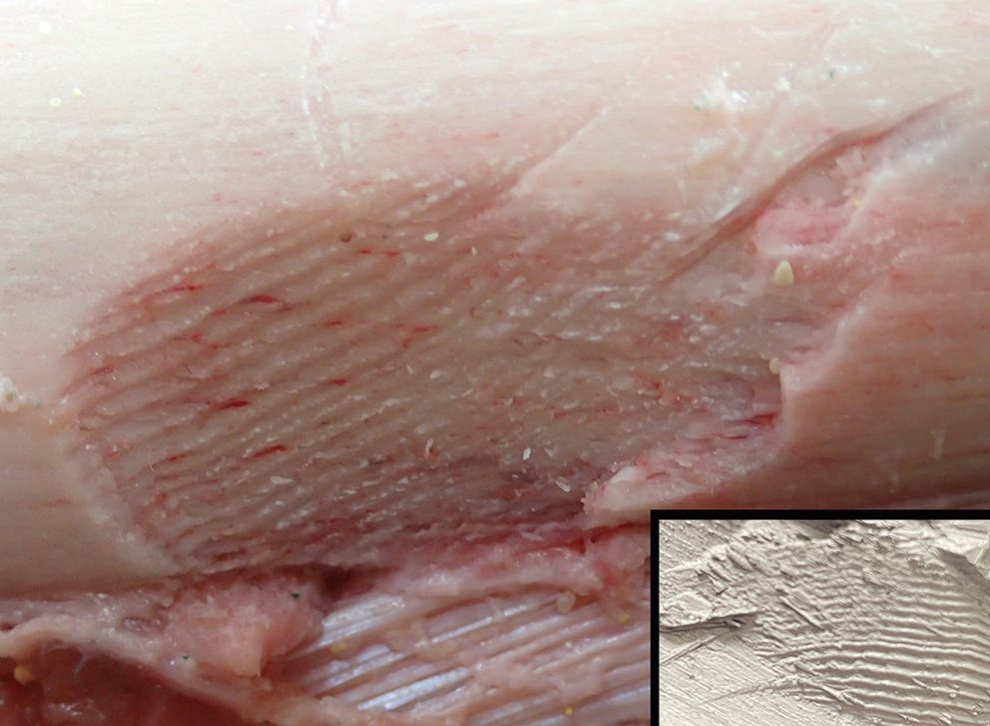
A shaved area of bone on the lateral aspect of the femur showing fine linear striations across its base from the serrated edge of the tooth of a white shark again with a silicone cast recording the features of the injury (Inset)

Approximately 300 mm of residual proximal femur remained with an irregular oblique fracture of the distal end and a clearly demarcated inverted ‘V’-shaped notch which measured 22 mm on the lateral side and 12 mm on the medial side (Figs. 2B and 3). The distance from the medial end to the lateral side was approximately 14 mm, with an angle of 54°. A Mikrosil® silicone cast was taken (Fig. 3). Immediately above the fracture line on the lateral aspect of the femur was an oblique superficial incised groove, running from right to left downward measuring approximately 20 mm. Medial to this there were three very superficial parallel incised grooves measuring 7 mm, 4 mm and 3 mm respectively. On the upper anterior aspect of the femur an oblique 15 mm incised groove was present running from right to left and downward. Below this and running from right to left and upward was a 19 mm superficial groove and below this a 20 mm superficial groove. The lowest bone injury in this area was a 30 mm incised groove running from right to left downward. In the central portion of the lower margin there was a series of fine cross-striations less than 1 mm apart. A Mikrosil® silicone cast was also taken of these (Fig. 4). On the lateral upper aspect of the femur a 25 × 15 mm scalloped defect that had been observed on CT images demonstrated numerous cross-striations in its base with less than 1 mm separation. A final Mikrosil® silicone cast was taken of these (Fig. 5). More proximal to this was another bony defect measuring 30 × 10 mm not associated with any striations. There were no injuries to the head, abdomen, chest or other limbs, apart from a minor bruise below the left knee. There were no abrasions to suggest that ‘bumping’ by the shark had occurred although this may have been masked by the interposition of the wet suit.

**Fig. 6:**
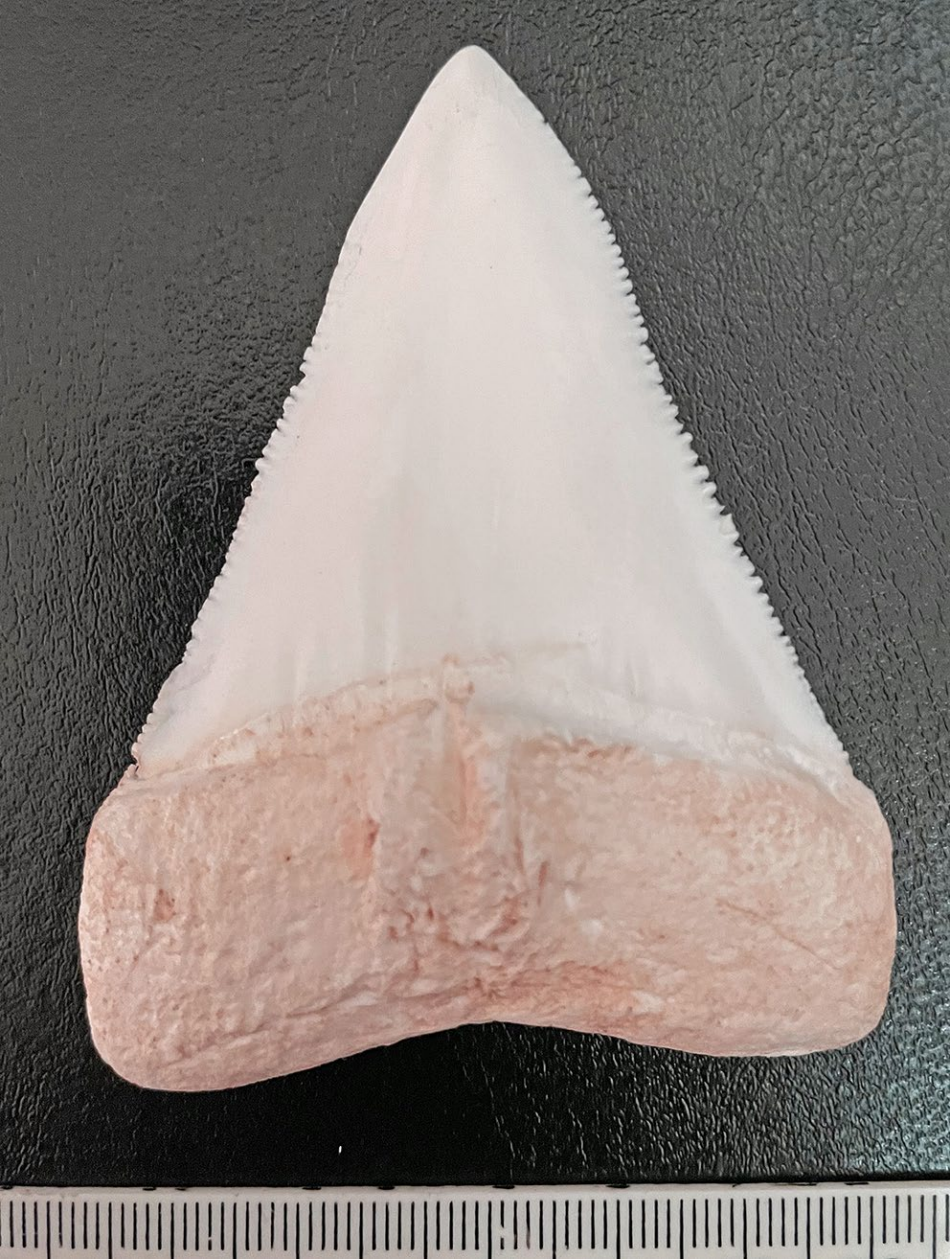
A cast of the tooth of a white shark showing marginal serrations that may cause linear striations similar to those observed in observed in Figs. 4 and 5

Death was therefore caused by exsanguination due to limb amputation from a shark attack. Other than the amputation and soft tissue removal, a number of the injuries were also typical of shark attack including the triangular-shaped wound of the skin of the right buttock, the ‘V’-shaped bony defect of the distal femur and the fine parallel striations from teeth serrations. A cast of a white shark tooth demonstrates characteristic marginal serrations (Fig. 6).

## Discussion

Although the white shark (also known as the great white shark) (*Carcharodon carcharias*) (which may reach lengths of 3.5 to 5.5 m) has a fearsome reputation, Australian data show that tiger shark (*Galeocerdo cuvier*) attacks are more likely to result in death. Specifically, while 38% of tiger shark and 32% of bull shark (*Carcharhinus leucas*) bites are fatal, only 25% of those due to white shark bites have resulted in death [11, 16]. Identification of the exact species after an attack may, however, sometimes be contentious [17] although both tooth morphology and calculated jaw shape may be useful [18]. Genetic investigations may also be used now to identify traces of shark DNA in the bite wounds of victims [19].

Injuries from shark attacks arise from a variety of mechanisms involving both blunt and sharp force trauma. Crushing and amputations occur due to the massive compressive forces from biting which in some species may reach up to 3300 kg/cm^2^ [20]. Penetrating and sharp force injuries result from a saw-like motion of the shark mouth with its rows of extremely sharp teeth [21] and a rolling or shaking movement of the head [22]. Problems in the evaluation of cases include failure to recover bodies, the damaging effects of putrefaction and post mortem animal feeding, and differentiating lethal wounds from those due to post mortem shark predation [14, 22–24]. In cases where a body has been literally torn apart by several sharks the only tissues available for examination may be aerated lung which has floated to the surface and subsequently been washed up along the shore [15].

Shark attacks vary from unproved to provoked, the latter where a shark may react to being approached in the water [4]. Unprovoked attacks may be so-called ‘bump and bite’ or ‘hit and run’ [25] possibly related to territorial instincts [13]. In the case of ‘bump and bite’ attacks a shark may knock into a victim before attacking which may leave characteristic abrasions from the rough scales (denticles) of the shark’s skin, unless there has been protection from a wet suit [13]. ‘Hit and run’ attacks are the most common, accounting for 80% of episodes and occurring usually in shallow water. This may be due to a swimmer being mistaken for a seal. In a ‘sneak’ attack a shark may attack unexpectedly without warning [25].

In the reported case, stripping of soft tissues from the right lower extremity below the groin was in keeping with the usual mode of attack with an approach from beneath [13]. It has been noted that 97% of victims have limb injuries [26]. The sharply incised edges of the wound without any abrasions and a triangular wound to the skin were typical of such events [13, 22]. Of interest, the triangular defect of the distal femur also paralleled this type of biting injury. The areas of shaving of the cortical bone demonstrated fine striations resulting from an oblique bite with dragging of the marginal serrations of the teeth of the shark. This enabled silicone castings to be made for permanent autopsy records. While serrations in bite marks may help to identify the species of shark involved, as in the current case, the type of serration is not unique to a particular animal and there are no predictable patterns in either their size or position; i.e. there can be no ‘teeth printing’ to identify a specific predator [16]. Although shark teeth are brittle and may chip or break off during an attack [22–25] no tooth fragments were identified in the reported case in bone or soft tissues, even with the application of a 3-dimensional volume rendering technique.

This case has demonstrated that high resolution post mortem CT scanning with 3-dimensional volume rendering visualisation in a case of fatal shark attack was able to clearly show the nature and number of the bony injuries. However, as the resolution was not high enough to demonstrate the fine parallel grooves from teeth serrations that were present at two sites, a formal pathological evaluation still complimented post mortem CT screening. While post mortem CT volume rendering technique did not identify fragments of teeth in the current cases it did provide a very useful technique to screen for such material particularly in residual traumatised soft tissues. Casting of bone lesions at autopsy may provide an additional record of a case.

## Key points

1. A 15-year-old male was attacked by a large white shark while surfing.
2. CT examination showed an above-knee amputation of the right lower limb with stripping of soft tissues.
3. 3-dimensional volume rendering revealed linear gouges, shaved cortical bone and an inverted ?V?-shaped defect at the distal margin of the femur.
4. At autopsy fine parallel cross-striations matching the marginal serrations of the teeth of a white shark were also identified.
5. Silicone casting provides a permanent record of bone lesions.

## Declarations

### Complete statement and declarations

The author wrote this paper.

There is no original data other than the case details.

Ethical approval has been given by Forensic SA Management.

The details are a matter of public record.

Author contribution - 100%
